# Apoptosis, Induced by Human α-Synuclein in Yeast, Can Occur Independent of Functional Mitochondria

**DOI:** 10.3390/cells9102203

**Published:** 2020-09-29

**Authors:** Damilare D. Akintade, Bhabatosh Chaudhuri

**Affiliations:** 1 School of Life Sciences, Medical School, University of Nottingham, Nottingham NG7 2UH, UK; 2 Leicester School of Pharmacy, De Montfort University, Leicester LE1 9BH, UK; BChaudhuri@dmu.ac.uk

**Keywords:** human α-synuclein, mitochondria-dependent, mitochondria-independent, yeast petites, yeast apoptosis

## Abstract

Human α-synuclein expression in baker’s yeast reportedly induces mitochondria-dependent apoptosis. Surprisingly, we find that, under de-repressing conditions of the inducible *MET25/GAL1* promoters, yeast cells expressing chromosomally-integrated copies of the human α-synuclein gene are not killed, but spontaneously form respiration-deficient rho-minus (ρ^−^) petites. Although yeast cells can undergo cell death (apoptosis) from loss of mitochondrial function, they can also survive without functional mitochondria. Such cells are referred to as ρ^0^ or ρ^−^ petites. This study reports that minimal expression of human α-synuclein in yeast, from *MET25*/*GAL1* promoter, gives rise to ρ^−^ petites. Interestingly, the full expression of α-synuclein, from the same promoters, in α-synuclein-triggered ρ^−^ petites and also in ρ^0^ petites (produced by treating ρ^+^ cells with the mutagen ethidium bromide) initiates apoptosis. The percentages of petites increase with increasing α-synuclein gene copy-number. ρ^−^ petites expressing α-synuclein from fully-induced *MET25*/*GAL1* promoters exhibit increased ROS levels, loss of mitochondrial membrane potential, and nuclear DNA fragmentation, with increasing copies of α-synuclein. Our results indicate that, for the first time in yeast, α-synuclein-triggered apoptosis can occur independently of functional mitochondria. The observation that α-synuclein naturally forms petites and that they can undergo apoptosis may have important implications in understanding the pathogenesis of Parkinson’s disease.

## 1. Introduction

The yeast *Saccharomyces cerevisiae* is an attractive tool for the elucidation of human cells’ diverse biochemical pathways, which includes mitochondria-dependent apoptosis, a form of programmed cell death [1,2,3]. It has been reported that apoptosis was induced in aged yeast cells by human α-synuclein (α-syn) overproduction; meanwhile, it was thought to cause Parkinson’s disease (PD) in human neuronal cells (PD) [4], occurs in the presence of functional mitochondria [5]. Moreover, in both yeast and human neurons, α-syn’s toxicity seems to be dependent on mitochondrial outer membrane regulator (VDAC) that controls the influx and efflux of metabolites in and out of the mitochondria [6].

Mitochondria, in ρ^+^ grande (i.e., normal) cells, are involved in respiration through oxidative phosphorylation. Ineffective mitochondrial oxidative phosphorylation can cause cellular stress in ρ^+^ cells leading to overproduction of ROS [7,8], which, in turn, can result in mitochondrial dysfunction [8]. Thus, rho-zero (ρ^0^) and rho-minus (ρ^−^) petites, cells that have lost their respiratory capacity, are formed. The ρ^0^ petites lack mitochondrial DNA (mtDNA), and therefore, have no mitochondrial function [9]. Although ρ^−^ petites contain mtDNA, deletions/mutations in their mtDNA cause mitochondrial dysfunction; also, mutations in nuclear genes, that affect mitochondrial function, are involved in the formation of ρ^−^ petites. Since Glycerol only allows respiratory growth, both ρ^0^ and ρ^−^ yeast petites cannot grow in cell culture medium containing Glycerol as the sole carbon source [10]. However, ρ^−^ yeast cells can be distinguished from ρ^0^ petites by the green-fluorescent dye SYTO18, which selectively stains yeast mtDNA [11].

Partial mitochondrial dysfunction, as seen in ρ^−^ yeast petites, is linked to the symptoms of Parkinson’s disease (PD) [12,13]. Ρ^−^ yeast cells also share greatly diminished activity of the mitochondrial electron transport chain with dopaminergic neurons of patients who have Parkinson’s disease (PD). Neuronal cell death in PD, as in α-syn-induced yeast apoptosis, occurs from complete loss of mitochondrial function [14,15].

A-syn, a presynaptic neuronal protein linked genetically and neuropathologically to PD [16], exists in a soluble monomeric form that is in equilibrium with its soluble oligomeric form, an insoluble fibrillar α-syn aggregate [17]. Although the exact physiological function of α-syn is not clear [18], α-syn aggregation constitute a significant factor in PD pathogenesis [19]. Through its mitochondria-targeting amino terminus that interacts with mitochondrial complex I function [18], wild-type and mutant α-syn overexpression can cause mitochondrial damage in neurons through the formation of intra-cytoplasmic fibrillar aggregates, known as Lewy bodies [20]. The α-syn A53T mutant protein, which is linked to early-onset PD, is much more prone to aggregation than the wild-type protein [21].

Growth of yeast cells in a medium that contains an mtDNA replication inhibitor and/or inhibitor of mitochondrial protein synthesis can result in partial or complete loss of mtDNA, giving rise to respiratory-deficient ρ^−^ and ρ^0^ petite yeast cells, respectively [22]. However, in human cells, the petite formation can occur spontaneously when mitochondrial function is partially disturbed by mtDNA mutations. This is the basis of most human neurological disorders [23]. Remarkably, artificially-created mtDNA-lacking human ρ^0^ cells [24], although more resistant to apoptosis than ρ^+^ cells, can still undergo cell death [25]. This is in contrast to the observation that cells with a deficiency in their respiratory chain may have increased apoptosis in vivo [26]. Interestingly, human ρ^−^ cells, partially depleted of mtDNA, preserve major apoptotic features, such as loss of mitochondrial membrane potential (MMP) and release of mitochondrial proteins [27]. Hence, it can be inferred that the execution of apoptosis can occur in human cells that have lost mtDNA partially or entirely.

In theory, yeast petites can be used to find out if, like human cells, they also can undergo apoptosis. There is a report indicating that ρ^+^ grande yeast cells treated with high concentrations of bleomycin do undergo mitochondria-independent apoptosis [28]. However, it seems that, in general, the presence of functional mitochondria is essential for yeast apoptosis induced by a variety of factors. These include heterologous expression of human proteins α-syn [29], and proapoptotic Bax of the Bcl-2-family [30], the process of cellular ageing [31], defects in actin function [32], acetic acid treatment of cells [33], and prolonged cell exposure to mating factors [34], plant toxins [35], osmotic stress [36], high pH environment [37], and lipid peroxidation [38]. Yeast cells facing stress from any of these factors, including that of α-syn expression, a prompt rise of intracellular ROS levels that lead to dysfunctional mitochondria, measured by a decrease in MMP, and ultimately to apoptosis [39,40,41]. In neuronal cells too, expression of α-syn causes an increase in ROS production, the opening of mitochondrial permeability transition pore, decrease in MMP, and eventual cell death [42].

This study describes the unexpected findings that very low levels of wild-type human α-syn expression in yeast, obtained through de-repression of the inducible *MET25* or *GAL1* promoter, spontaneously cause the formation of ρ^−^ petites, which undergo apoptosis when the promoters are fully induced (i.e., when higher levels of α-syn are produced). Even in the absence of mtDNA, α-syn-expressing ρ^0^ cells, generated by treating with the chemical mutagen ethidium bromide, undergo nuclear DNA fragmentation (a hallmark of apoptosis). This is the first time that it is being described that, in the absence of functional mitochondria, α-syn can trigger apoptosis in yeast cells. It should be noted that ρ^−^ petites are not formed in yeast cells minimally expressing the A53T mutant α-syn [21], but instead, cells directly undergo apoptosis (data not shown). The results presented here could have broader implications in understanding the pathogenesis and pathophysiology of Parkinson’s disease.

## 2. Materials and Methods

### 2.1. Yeast Strains

The yeast strain W303-1A Mata (ATCC #208352), referred to in this study as BC300 and is auxotrophic for the genes *ADE2*, *HIS3*, *LEU2*, *TRP1* and *URA3*. New yeast strains were derived from BC300 by transforming integrative plasmids (Supplementary Materials, Parts 1 and 2), which would express α-syn from the *MET25* or *GAL1* promoter. The ρ^0^ petites were obtained from the ρ^+^ (grande) BC300 strain through ethidium bromide mediated loss of mtDNA, using a published protocol [43]. In brief, BC300 was grown in full YPD liquid medium containing 4% glucose and ethidium bromide (20 µg/mL) overnight. The harvested cells were mostly ρ^0^; they grew on YPD, but not on YP-glycerol plates, and did not contain mtDNA.

### 2.2. Yeast Transformation

Plasmids bearing α-syn gene expression cassettes under the control of either the methionine-repressible *MET25* or galactose-inducible *GAL1* promoter (*MET25*p/*GAL1*p; see Supplementary Materials, Parts 1 and 2) were used for genomic integration at the *TRP1*, *HIS3*, and *URA3* chromosomal loci of the ρ^+^ BC300 strain to yield strains that contain 1–3 copies of α-syn. The integrative transformation was carried out using a published protocol [44]. Similarly, a ρ^0^ BC300 strain was sequentially transformed with integrative plasmids bearing only the *MET25*p-α-syn expression cassettes to obtain ρ^0^ strains that contain 1–3 copies of α-syn. The table is shown in Part 6 (Supplementary Materials).

### 2.3. SYTO18 Staining of mtDNA

SYTO18 (Molecular Probes; #Y7530) is a green fluorescent dye that selectively stains yeast mtDNA [45,46]. After the expression of α-syn in yeast, ~1 × 10^6^ cells were suspended in 1 mL of 10 mM HEPES buffer (pH 7.4; containing 5% glucose). A 10 µM final concentration of SYTO18 was added to cells, which were then incubated at room temperature for 5 min. Cells were pelleted and re-suspended in fresh 10 mM HEPES buffer (pH 7.4; containing 5% glucose). Fluorescent cells were visualized using a fluorescence microscope.

### 2.4. Detection of Dead Cells with Phloxine B Dye

Cell death was assessed by staining cells with the red dye Phloxine B (Sigma, Dorset, UK. P-4030-25G) [47]. Live cells expel the dye, whereas it is accumulated in dead cells. This can be observed by fluorescence microscopy. Staining experiments were performed exactly as published earlier [48].

### 2.5. Detection of ROS

AAT Bioquest (Sunnyvale, CA, USA) Fluorimetric Intracellular Total ROS Activity Assay Kit (#22901) was used for measuring ROS. Experiments were performed as published earlier [48].

### 2.6. Assessing the Presence/Absence of Mitochondrial Membrane Potential (MMP) by Staining Live Cells with the Dye DiOC6(3)

The DiR dye (AAT Bioquest; Sunnyvale, CA, USA #22046), belonging to the DiOC_6_(3) (3,3′-dihexyloxacarbocyanine iodide) family of fluorescent stains, is a deep red lipophilic agent that accumulates in mitochondria of live cells when used at low concentrations. It is used to monitor mitochondrial membrane potential (MMP). The DiR/DiOC_6_(3) dye was used to confirm the presence or absence of MMP in (visually^−^ and ρ^0^ yeast petites. After cells were grown for expression of α-syn, OD600 was measured, and cells were counted. 1 × 10^6^ cells were suspended in 1 mL of 10 mM HEPES buffer, containing 5% glucose, at pH 7.4. A final concentration of 175 nM of DiOC_6_(3) was added to cells and incubated at room temperature for 13 min. Imaging was done with an Olympus fluorescence microscope (Essex, UK) (at excitation/emission 484/501 nm) with a Leica digital imaging camera.

### 2.7. Quantifying Mitochondrial Membrane Potential (MMP) with the JC-10 Dye

AAT Bioquest (Sunnyvale, CA, USA) JC-10 Mitochondrial Membrane Potential Assay kit (#22800) uses water-soluble JC-10 to determine MMP quantitively. Experiments were conducted as per the published protocol [48].

### 2.8. Staining with Hoechst Dye for Monitoring Live Cells

Hoechst 33,258 (Thermo Fisher Scientific, Loughborough, UK; #H21491) is a nucleic acid stain widely used to detect live cells. When bound to double-stranded DNA, the dye emits blue fluorescence. Staining with the dye was performed as described earlier [48].

### 2.9. Assessing Nuclear DNA Fragmentation via the TUNEL Assay. AA

AAT Bioquest (Sunnyvale, CA, USA) TUNEL Apoptosis Assay kit (#22844) was used for the detection of nuclear DNA fragmentation (NDF). The assays were performed as described earlier [48].

### 2.10. Western Blotting

Western blotting was carried out using standard protocols [49], using primary antibodies specific to α-syn (Proteintech; Manchester, UK #10842-1-AP) or β-actin (Proteintech; Manchester, UK #60008-1-Ig).

### 2.11. Quantification of Petite formation

After transformation, colonies of transformants were screen on YP-Gly (with Glycerol as the carbon source). Moreover, after the growth of yeast cell under de-repressing condition, 200 cells were spread on a YPD plate, colonies were then screened on the YPGly agar plate. In both cases, cells that grew (Grande cells) on YPGly were counted, and cells that did not grow (Petite) on YPGly were also counted.

## 3. Results and Discussion

### 3.1. Minimal Expression of Human α-Syn in Yeast from the MET25 or GAL1 Promoters (MET25p or GAL1p), under De-Repressing Conditions, Produces Petites

**Figure 1:**
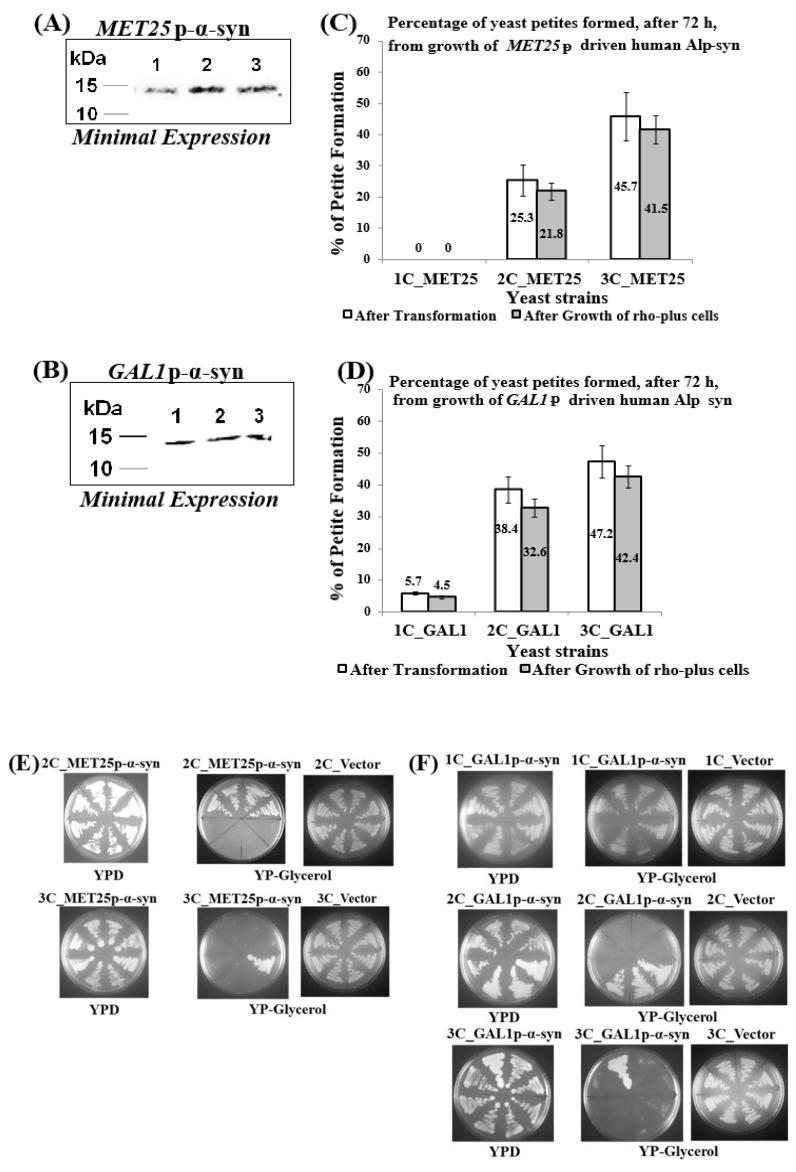
Petites obtained after minimal expression of α-syn from the *MET25* or *GAL1* promoter. (**A**,**B**) Western blot analyses of cells expressing 1 to 3 copies of α-syn protein from either *MET25*p (**A**) or *GAL1*p (**B**) The blots were probed with an antibody that recognizes α-syn protein (Proteintech, 10842-1-AP); (**C**,**D**) The percentage of petites formed (in cells containing 1–3 copies of α-syn under the control of *MET25*p/*GAL1*p) from a total of 50 transformants (non-shaded bars) or in 200 cells obtained. The data represent the mean ± S.D. of three independent experiments. Post Hoc Newman-Keuls test was carried out after a significant ANOVA test, indicating a significant difference *p* < 0.01 in petites formed between cells expressing 1opy and 2 or 3-copies of α-syn. (**E**,**F**) Representative pictures of growth of yeast cells harboring 1-copy (1C), 2-copies (2C), and 3-copies (3C) of the human α-syn gene, under the control of *MET25*p (**E**) and *GAL1*p (**F**), on complete medium solid agar plates that contained either glucose (YPD; plates 1) or Glycerol (YP-glycerol; plates 2). ‘Vector’ represents cells that contain empty plasmid(s), with no α-syn gene, grown on YP-glycerol (plates 3).

During the process of sequential chromosomal integration of *MET25*p or *GAL1*p-driven human α-syn gene-expression cassette-bearing plasmids (Supplementary Materials, Parts 1 and 2) to obtain yeast strains containing 1, 2, and 3 copies of the α-syn gene, it was found, after 72 h incubation at 30 °C, that a percentage of transformants did not grow on solid-agar complete YP medium plates that contained Glycerol as the sole carbon source (Figure 1C,D; non-shaded bars; the only exception, where all transformants grew on Glycerol, were cells containing 1-copy *MET25*p-α-syn). This suggested that transformants that did not grow on Glycerol could not respire aerobically (i.e., were respiration-deficient), as Glycerol is strictly a respiratory carbon source. When MET25p or GAL1p is de-repressed, α-syn protein is minimally expressed. Figure 1A,B show levels of α-syn protein present in 100 µg of cell lysate.

Hence, it was inferred that they were petites. It was, therefore, decided to grow normal, non-petite, ρ^+^ transformants under conditions that allow de-repression of the promoters to see if α-syn-bearing petites were formed spontaneously.

For strains containing 1–3 copies of the α-syn gene under the control of the *MET25*p, cells were grown for 72 h in glucose-containing SD minimal medium that contained 675 µM methionine for repression of *MET25*p; over a 72-h growth period, in the presence of methionine, there may be mild de-repression of the *MET25*p because the methionine concentration used to repress the promoter may not entirely block transcription of the downstream α-syn gene [50]. For strains containing 1–3 copies of the α-syn gene under the control of the *GAL1*p, cells were grown for 72 h in SD medium; under these conditions, in the presence of ethanol, which is produced upon complete conversion of glucose to ethanol after 12–13 h of cell growth, *GAL1*p is de-repressed [51].

**Figure 2:**
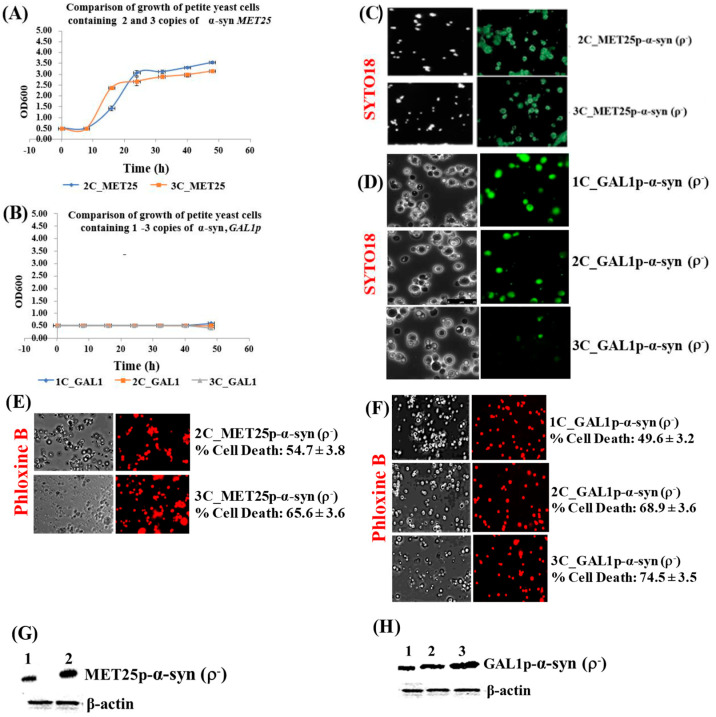
(**A**,**B**) Comparison of the growth curves of ρ^−^ cells expressing 2–3 copies of α-syn from *MET25p* (**A**) and 1–3 copies of α-syn from *GAL1p* (**B**); cells were grown in YPD (**A**) and YP-galactose (**B**). Bonferroni post hoc test after a significant two-way ANOVA indicates no significant difference in growth between yeast petites that contain different copies of the α-syn gene. (**C**,**D**) Microscopic images (×400) of petite cells, where expression of 2–3 copies (*MET25p*) or 1–3 copies (*GAL1p*) of α-syn gene was induced, stained with the dye SYTO18. (**E**,**F**) Microscopic images (×400) of petite cells expressing 2–3 copies of α-syn from the *MET25p* (**E**) and 1–3 copies of α-syn from the *GAL1p*, staining with Phloxine B. (**G**,**H**) Western blot analyses of cells expressing 1 to 3-copies of α-syn protein after full induction of the *MET25p* (**G**) or *GAL1p* (**H**). On lanes, 1, 2 (**G**) and 1, 2 and 3 (**H**) were loaded 7.5 µg of total protein obtained after lysis of cells that express 2-copies or 1-copy (lanes 1; **G**,**H**), 3-copies or 2-copies (lanes 2; **G**,**H**) and 3-copies (lane 3; **H**) of α-syn, after the growth of cells under conditions that fully induce the MET25 or GAL1 promoter. The upper panel was probed with an antibody that recognizes human α-syn (Proteintech, #10842-1-AP) and the lower panel with a β-actin antibody (Proteintech, 60008-1-Ig); Densitometric quantification of the α-syn bands in (**G**,**H**) is shown in Supplementary Materials, Parts 3 and 4. Post Hoc Newman-Keuls test, after a significant one-way ANOVA test, indicated a significant difference in cell death, *p* < 0.01, between petites expressing α-syn with different copy numbers.

This is in contrast to the much higher levels of α-syn protein obtained from fully induced *MET25*p/*GAL1*p where only 7.5 ug of cell lysates were used for Western blots (Figure 2G,H).

The percentages of petites formed in liquid culture, under de-repressing conditions of growth of cells containing human α-syn gene downstream of the *MET25*/GAL1 promoters (Figure 1C,D; shaded bars), were confirmed by replica-plating cells from complete YPD-agar plates (that contained glucose; plates 1 in Figure 1E,F) on to YP-glycerol agar plates (that contained Glycerol; plates 2 in Figure 1E,F). Cells that do not grow in Glycerol are the petites, and the ones that do grow in Glycerol are ρ^+^ grande cells. Plates 3 (in Figure 1E,F) display cells containing empty plasmids (i.e., that bear no α-syn gene), which show that petites are not formed in the absence of α-syn. Moreover, as observed during transformation, petites are not formed in cells expressing 1-copy *MET25*p-α-syn (results not shown). The petite percentages formed in liquid minimal medium culture are similar to the percentages of transformant colonies that were identified as petites after integrative transformation of the basic ρ^+^ yeast strain BC300 (i.e., W303-1A) with the α-syn-bearing plasmids (compare shaded and non-shaded bars in Figure 1C,D).

### 3.2. Expression, in ρ^−^ Yeast Petites, of Human α-Syn Gene from Fully-Induced MET25p/GAL1p Retards Cell Growth and Causes Cell Death

The promoters *MET25*p and *GAL1*p were then induced so that α-syn could be fully expressed in the petites that had been formed by minimal expression of α-syn. The full expression of α-syn from the *MET25*p occurs in glucose-containing complete YPD medium without the addition of methionine. In contrast, galactose-containing complete YP-galactose medium fully induces α-syn expression from the *GAL1*p. Growth of petite cells (which did not grow in YP-glycerol medium; Figure 1E,F) fully expressing α-syn in liquid YPD or YP-galactose medium were monitored over 50 h at 30 °C, 180 rpm. There was no growth in petites expressing α-syn from the *GAL1*p (Figure 2B), whereas for petites expressing α-syn from the *MET25*p, there was initial growth which plateaued off after 24 h (Figure 2A). It was confirmed that petite cells, grown in an appropriate expression medium for 50 h, contained mtDNA by staining with SYTO18, a green fluorescent dye that selectively stains yeast mtDNA (Figure 2C,D) [45], indicating that the petites were ρ^−^. Staining with Phloxine B, which stains only dead cells red [47], showed that α-syn-expressing ρ^−^ cells underwent cell death (Figure 2E,F). It was observed that cell death gradually increases with increasing α-syn copy-number. This mirrored increasing levels of the α-syn protein produced in cells expressing 1–3 copies of α-syn, from the *MET25* or *GAL1* promoter (Figure 2G,H; also see densitometric quantification of α-syn bands in Supplementary Materials, Parts 3 and 4).

### 3.3. After Full Induction of MET25/GAL1 Promoter, Human α-Syn Expressing ρ^−^ Yeast Petites Undergo Loss of MMP and Increase in Nuclear DNA Fragmentation upon Gradual Increase of α-Syn Copy Number from 2–3 or 1–3 Copies

**Figure 3:**
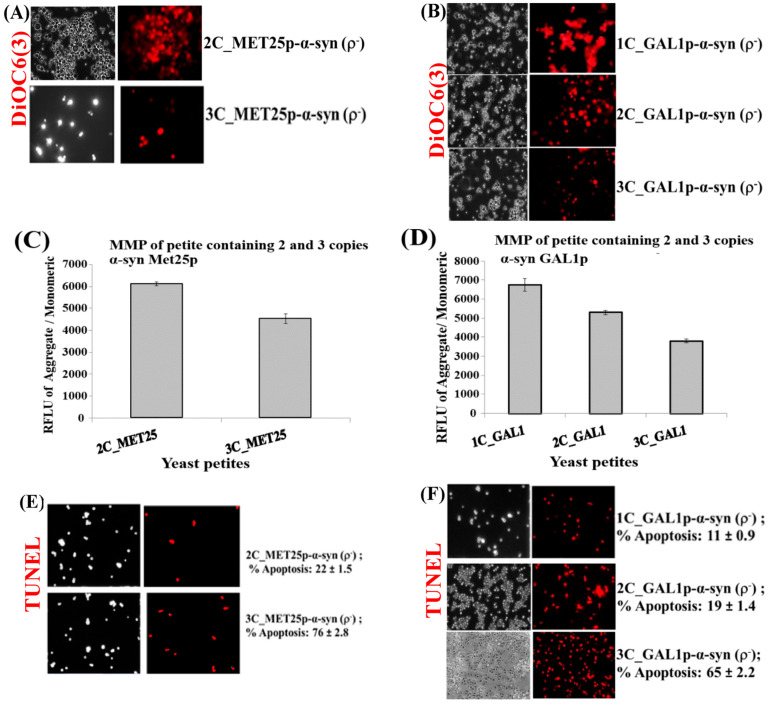
(**A**,**B**) Microscopic images (×400) of staining of 3 copies of α-syn petite transformants with DiOC6(3) dye that detects MMP in live cells. The images are representative images (×400) of cells. (**C**,**D**) Quantification of relative MMP of yeast cells expressing 2 and 3-copies of α-syn from *MET25p* and 1–3-copies of α-syn from *GAL1p*, using the JC-10 dye (*p* < 0.1). (**E**) Microscopic images (×400) of nuclear DNA fragmentation as observed using the TUNEL assay in yeast cells expressing 2 and 3 copies of α-syn from *MET25p*. (**F**) Microscopic images (×400) of nuclear DNA fragmentation as observed using the TUNEL assay in yeast cells expressing 1 copy of α-syn from *GAL1p*. The images are representative images of cells. The data in figures (**C**,**D**) represent mean ± S.D. of three independent experiments (*p* < 0.1; two-tailed *t*-test). The left-hand side pictures in (**A**,**B**,**E**,**F**) show phase-contrast microscopy pictures (×400) of yeast cells. Post Hoc Newman-Keuls test, after a significant one-way ANOVA test, indicated a significant difference *p* < 0.001 between strains expressing 2 and 3-copies of α-syn from *MET25p*, and between 1 and 2-copies and 3-copies of α-syn from *GAL1p*.

The integrity of mitochondrial membranes, within α-syn expressing ρ^−^ yeast petites, were then monitored using the red fluorescent DiR dye, a variant of the dye 3,3′-dihexyloxacarbocyanine iodide, DiOC6(3), which stains functional mitochondrial membranes [5]; see Figure 3A,B.

The results showed weaker staining of mitochondria in ρ^−^ petites that expressed 2 and 3 copies of α-syn from *GAL1*p than cells expressing 1-copy of α-syn from *GAL1*p. Similarly, mitochondria in cells expressing 2-copies of α-syn from *MET25*p were more strongly stained than cells expressing 3-copies of α-syn from *MET25*p. This would indicate that cells expressing lesser copies of α-syn were more alive, with intact mitochondrial membranes, than cells that expressed more copies of α-syn. This was corroborated by data obtained after quantification of mitochondrial membrane potential (MMP) [52], on a fluorescence plate reader, using the JC-10 MMP assay kit (Abcam; Figure 3C,D). It confirmed that ρ^−^ petites, formed upon minimal expression of α-syn, when expressing 1 or 2-copies of α-syn have higher MMP compared to petites that expressed 2 or 3-copies of α-syn. This reflected increased toxicity within cells as α-syn copy number increased, and also indicated that the 3-copy α-syn strain suffered the most mitochondrial dysfunction.

Ρ^−^ petite cells, which underwent loss of MMP with increasing α-syn copy number, were then assessed via TUNEL assay to determine whether nuclear DNA fragmentation, a hallmark of apoptosis [53], occurred in these cells. The results clearly showed that ρ^−^ petites that suffered most apoptosis were the cells that expressed 3-copies of α-syn from *MET25*p or *GAL1*p (Figure 3E,F). It was observed that one copy of α-syn under the control of Met25 did not produce petite, and also from Figure 2A,B, α-syn was seen to be more toxic under the control of GAL1p. This observation could be due to the promoter effect. The Peptone and yeast extract complex substrates (in YPD medium) contain amino acids to support growth; this includes Methionine, which represses Met25p. Although we saw cell death after Met25 induction, YPD contains low indefinite amounts of Methionine, and this could affect the strength of the Met25 promoter, and hence, α-syn toxicity. The difference in α-syn toxicity between the two promoters (GAL1p and Met25p) could be due to the above condition.

### 3.4. Yeast Petite Cells Generated from ρ^+^ Basic Yeast Strain Do Not Contain mtDNA or Functional Mitochondrial Membranes

**Figure 4:**
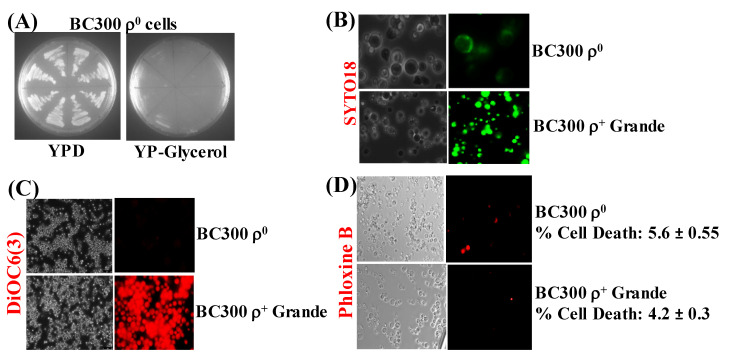
(**A**) Representative pictures of growth of ρ^0^ BC300 yeast cells, formed after ethidium bromide treatment, on complete medium solid agar plates that contained either glucose (YPD) or Glycerol (YP-glycerol). (**B**) Microscopic images (×600) of ρ^0^ petite and ρ^+^ grande BC300 cells stained with the fluorescent dye SYTO18, which selectively stains yeast mtDNA. (**C**) Staining of ρ^0^ petite and ρ^+^ BC300 cells with the dye DiR/DiOC6(3) that detects MMP in live cells (×400). (**D**) Microscopic images (×400) of ρ^0^ petite and ρ^+^ BC300 cells stained with Phloxine B. Post Hoc Newman-Keuls test after a significant one-way ANOVA test, indicated a significant difference *p* < 0.001 between ρ^0^ petite and ρ^+^ BC300 strains in experiments (**B**,**C**), whereas, statistically, there was no difference between ρ^0^ petite and ρ^+^ cells in (**D**).

ρ^−^ petites were spontaneously formed during minimal expression of α-syn in ρ^+^ cells. We then decided to find out the consequence of expressing α-syn in a chemically-induced respiration-deficient ρ^0^ yeast strain. Hence, ρ^+^ BC300 grande cells were treated with ethidium bromide to create strains devoid of mtDNA [43]. The cells obtained after ethidium bromide treatment were at first clonally selected to identify cells that did not respire (i.e., cells that did not grow on Glycerol). The growth of eight such clones, on agar plates, is shown in Figure 4A.

The cells from one of these clonally-selected strains were then stained with SYTO18 and DiOC6(3), which showed complete absence of mtDNA and functional mitochondrial membranes, respectively (Figure 4B,C), suggesting that the strain was a ρ^0^ derivative of BC300. The ρ^0^ and ρ^+^ grande cells displayed no difference after staining with Phloxine B, indicating that, besides lacking mtDNA and functional mitochondrial membranes, the ρ^0^ cells are as alive as the ρ^+^ cells (Figure 4D).

### 3.5. ρ^0^ Yeast Petite Cells, that Bear MET25p-α-Syn Integrative Plasmids, on Expression of α-Syn Undergo Cell Death and Nuclear DNA Fragmentation (Apoptosis)

The ρ^0^ BC300 strain was then sequentially transformed with *MET25*p-α-syn bearing integrative plasmids (Supplementary Materials, Part 1) to obtain strains that contained 1–3 copies of α-syn. The *MET25*p-driven α-syn integrative plasmids were chosen over the *GAL1*p-driven α-syn integrative plasmids mainly because of the full expression of α-syn from *MET25*p can occur in a glucose-containing medium. In contrast, expression from *GAL1*p requires medium containing galactose, which allows growth primarily under respiratory conditions [54]. Since ρ^0^ cells completely lack respiratory capacity, we thought it would be inadvisable to use the *GAL1*p constructs.

**Figure 5:**
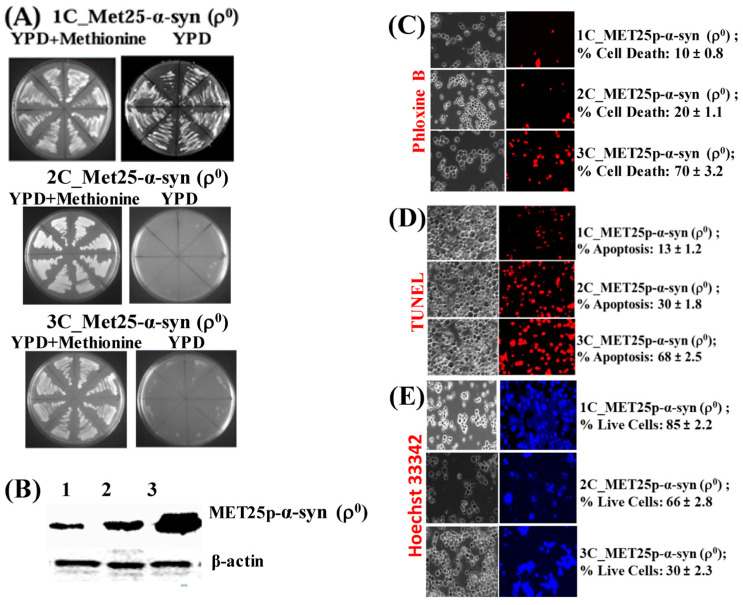
(**A**) Image of growth of Ῥ0 yeast cells harboring 1 copy (1C) of the α-syn gene under *MET25p* control on complete YPD medium agar plates in the presence (YPD + Methionine) or absence of methionine. (**B**) Western blot analyses of ρ^0^ cells expressing 1-copy (lane 1), 2-copies (lane 2), and 3-copies (lane 3) of α-syn protein. On lanes 1, 2, and 3 were loaded 7.5 µg of total protein obtained after lysis of cells. The upper panel was probed with an antibody that recognizes the HA epitope (Proteintech, 51064-2-AP) and the lower panel with a β-actin antibody (Proteintech, 60008-1-Ig); levels of β-actin were used as loading controls, β-actin being a housekeeping gene. (**C**) Microscopic images (×400) of ρ^0^ cells, stained with Phloxine B, after the expression of 1–3 copies of α-syn from the *MET25p*. (**D**) Microscopic images (×400) of nuclear DNA fragmentation, as observed using the TUNEL assay, in ρ^0^ yeast cells expressing 1–3-copies of α-syn from *MET25p*. (**E**) ρ^0^ cells, bearing 1–3 copies of *MET25p*-driven α-syn expression cassettes, were stained with Hoechst 33,342 (a blue dye that labels DNA of live cells). Post Hoc Newman-Keuls test after a significant one-way ANOVA test, indicated a significant difference between petites expressing 1–3 copies of α-syn.

The ρ^0^ yeast strains containing *MET25*p-driven α-syn expression cassettes in 1–3 copies were used to find out if they could undergo cell death, and more specifically, apoptosis when α-syn was fully expressed from the *MET25* promoter, namely, in complete YPD medium lacking methionine. At first, the ρ^0^ transformants were grown on YPD agar plates for 96 h at 30 °C (Figure 5A).

The ρ^0^ cells containing 1-copy of α-syn grew upon expression of the protein on a YPD plate. Cells containing 2 and 3 copies, however, did not grow, implying that comparatively lower levels of α-syn produced from 1-copy gene expression cannot block cell growth on plates. The expression of α-syn in the 1–3 copy α-syn-containing ρ^0^ cells was then assessed via Western blotting, after growing cells in YPD liquid medium for 50 h at 30 °C (Figure 5B). Densitometric quantification showed that the 3-copy strain expressed the highest amount of α-syn (see Supplementary Materials, Part 5). The same 1–3 copy α-syn-containing ρ^0^ cells, grown in YPD liquid medium, were then stained with Phloxine B to determine the percentage of cell death (Figure 5C). After that, the cells were tested for nuclear DNA fragmentation using the TUNEL assay (Figure 5D). There was a remarkable increase in cell death from 1-copy to 3-copy α-syn expressing cells, as seen in both Phloxine B and TUNEL assays (Figure 5C,D). In parallel, the cells were stained with the blue fluorescent dye Hoechst 33,342 (Figure 5E), which stains the DNA and nuclei of live cells [55]. It was clearly seen that there were far more live cells in the 1 and 2-copy α-syn-containing ρ^0^ cells than in cells expressing 3-copies of α-syn.

### 3.6. Like ρ^+^ Cells, ρ^0^ and ρ^−^ Cells Undergo Apoptosis When α-Syn is Expressed from the MET25 Promoter in Complete YPD Medium

**Figure 6:**
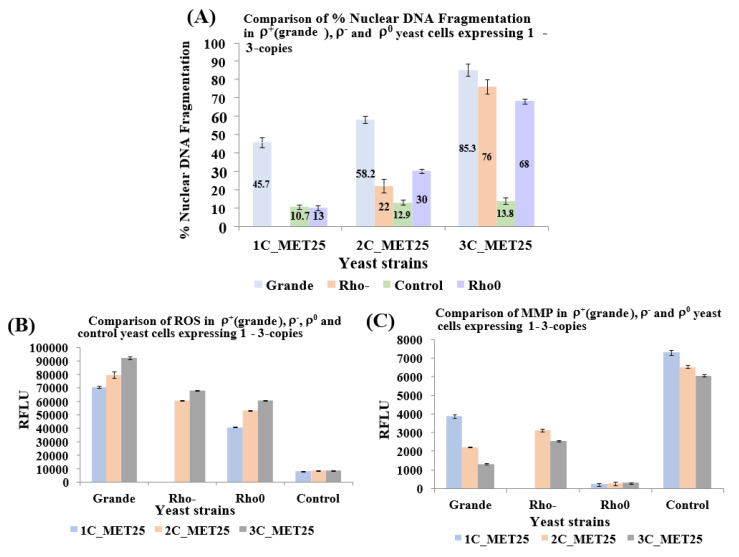
Analysis of nuclear DNA fragmentation (**A**), an increase of reactive oxygen species (ROS; **B**), and loss of mitochondrial membrane potential (MMP; **C**), in ρ^0^ yeast cells that bear 1 to 3-copies of the human α-syn, gene under the control of the *MET25* promoter. (**A**) Levels of nuclear DNA fragmentation, a hallmark of apoptosis, in yeast cells that express 1 to 3-copies of α-syn were assessed using the TUNEL assay. (**B**) Cells were stained with dihydroethidine to detect ROS. (**C**) Cells were stained with the JC-10 dye to quantify MMP loss. The data presented in (**A**,**B**,**C**) represent mean ± S.D. of three independent experiments (*p* < 0.05).

The TUNEL assay was used to compare nuclear DNA fragmentation (NDF), a hallmark of apoptosis, that was seen to occur in ρ^+^, ρ^−^ and ρ^0^ cells expressing α-syn from *MET25*p. In all cells, plasmids bearing α-syn expression cassettes had been integrated at different chromosomal loci (at *HIS3* for 1-copy, *HIS3*, *URA3* for 2-copies, and *HIS3*, *URA3*, *TRP1* for 3-copies). All recombinant cells were grown in YPD (lacking methionine) for 20 h at 30 °C for full induction of the *MET25*p. The comparative results are presented in Figure 6A and show that levels of NDF in ρ^+^, ρ^−^ and ρ^0^ cells expressing 3-copies of α-syn are similar.

Since the expression of human α-syn in baker’s yeast results in the accumulation of ROS, followed by the manifestation of apoptosis [56], ROS levels were measured (Figure 6B). Results showed that α-syn generates ROS both in ρ^−^ and ρ^0^ cells, as in ρ^+^ cells. MMP was also measured in all cell types (Figure 6C) because α-syn aggregation has been reported to cause mitochondrial dysfunction, thereby affecting MMP [41]. The results with ρ^0^ cells stood out; MMP levels were minuscule since ρ^0^ cells do not contain any mitochondria (was shown not to stain with SYTO18 and DiOC6(3), as shown in Figure 4B,C). The JC-10 dye concentrates in the mitochondrial matrix-forming red fluorescent aggregates. In apoptotic cells, JC-10 diffuses out of mitochondria and changes to a monomeric form, which emits green fluorescence. However, cells that do not contain mitochondria (i.e., ρ^0^ cells), JC-10 cannot concentrate on the mitochondria at all. The ratio of aggregated/monomeric JC-10 reflected that (Figure 6C). In hereditary genetics, inherited mitochondrial defects linked to mitochondrial dysfunction have been described in neurodegenerative disorders [57]. As of yet, the relationship between PD and mitochondrial dysfunction is unclear [21]. Polymorphisms related to mitochondrial genes are involved both in PD and also in Alzheimer’s disease (AD). PD is explicitly associated with oxidative stress and mitochondrial dysfunction in neurons of the substantia nigra, which ultimately leads to neurodegeneration [58,59]. However, this report is inconsistent with experiments published elsewhere [60]. Partial mitochondrial dysfunction, as seen in rho- yeast petites is linked to the symptoms of Parkinson’s disease (PD) [12,13]. Neuronal cell death in PD, as in α-syn-induced yeast apoptosis, occurs from complete loss of mitochondrial function [14,15]. However, in human cells, the petite formation can occur spontaneously when mitochondrial function is partially disturbed by mtDNA mutations. This is the basis of most human neurological disorders [23]. Remarkably, artificially-created mtDNA-lacking human rho0 cells, although more resistant to apoptosis than rho+ cells, can still undergo cell death [25]. This is in contrast to the observation that cells with a deficiency in their respiratory chain may have increased apoptosis in vivo [26].

## 4. Conclusions

Here, we show that human α-syn, when minimally expressed in yeast, has the unusual ability to form rho-petites. The results obtained after full expression of α-syn in rho- and rho0 yeast cells showed some death in the petite cells; this indicates that mitochondrial function is not an absolute requirement for α-syn-mediated apoptosis. The spontaneous formation of rho-petites by low-level expression of human α-syn in yeast could help in understanding the pathogenesis of Parkinson’s disease (PD) and its pathophysiology. Human cells usually cannot survive with a total loss of mitochondrial function, implying that impairment in mammalian cell mitochondrial, could result in apoptosis (cell death). Petite formation was not seen in mutant α-syn (A53 and A30P) in yeast. One could imagine that neuronal cells in PD initially suffer from the petite formation, which later undergoes death (i.e., neurodegeneration), as seen in PD, during the progression of the disease. α-syn-expressing yeast cells undergo a dose-dependent transit from mitochondrial loss to cell death. Therefore, high levels of alpha-syn are still able to induce cell death in cells that have previously lost mitochondrial respiratory function. Hence, functional mitochondria may be dispensable, but the loss of mitochondrial function may be essential for alpha-syn-induced cell death, as Partial mitochondrial dysfunction has been linked to the symptoms of Parkinson’s disease (PD).

## Supplementary Materials

The following are available online at https://www.mdpi.com/2073-4409/9/10/2203/s1. **Part 1**: Construction of Yeast Strains that Contain 1-Copy, 2- And 3-Copies Of The Human α-Syn Gene Driven by the MET25 Promoter (MET25P); **Part 2**: Construction of Yeast Strains that Contain 1-copy, 2- and 3-Copies of the Human α-Syn Gene Driven by the *GAL1* Promoter (*GAL1*p); **Part 3**: Densitometric Quantification of Western Blot Protein Bands after the expression of 2- and 3-Copies of α-Syn Gene from *MET25*p, in ρ^−^ Petite Yeast Cells; **Part 4**: Densitometric Quantification of Western Blot Protein Bands after the expression of 2 and 3-Copies of α-Syn Gene from *GAL1*p, in ρ^−^ Petite Yeast Cells; **Part 5**: Densitometric Quantification of Western Blot Protein Bands after the expression of 1, 2 and 3-Copies of α-Syn Gene from *MET25*p, in ρ^0^ Petite Yeast Cells; **Part 6**: Strain table.
