# Correction: Akintade, D.D.; Chaudhuri, B. Apoptosis, Induced by Human α-Synuclein in Yeast, Can Occur Independent of Functional Mitochondria. *Cells* 2020, *9*, 2203

**DOI:** 10.3390/cells14151128

**Published:** 2025-07-22

**Authors:** Damilare D. Akintade, Bhabatosh Chaudhuri

**Affiliations:** 1 School of Life Sciences, Medical School, University of Nottingham, Nottingham NG7 2UH, UK; 2 Leicester School of Pharmacy, De Montfort University, Leicester LE1 9BH, UK; BChaudhuri@dmu.ac.uk

In the original publication [1], due to figure overlap the Figures 2, 3 and 5 were revised.

## Error in Figure 2

There was a mistake in Figure 2C in the white light image.

**Figure 2:**
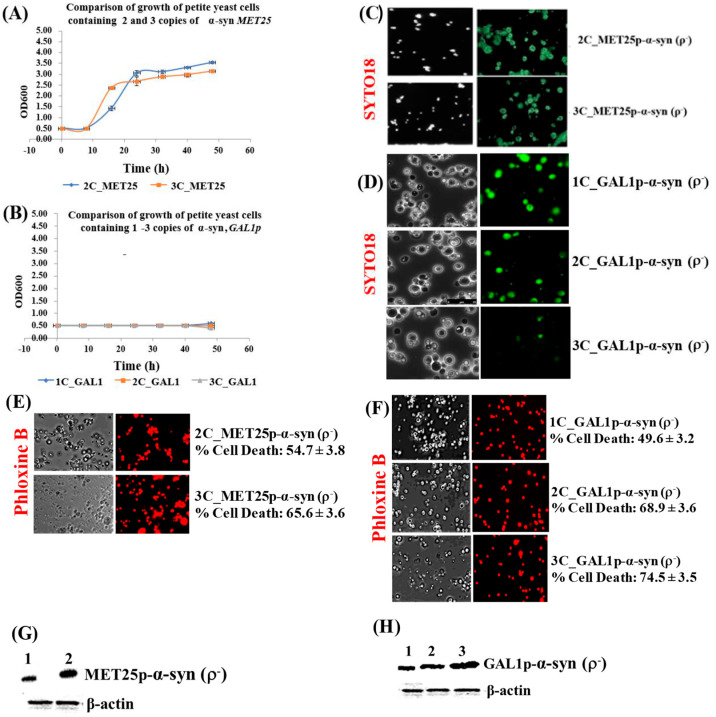
(**A**,**B**) Comparison of the growth curves of ρ^−^ cells expressing 2–3 copies of α-syn from *MET25p* (**A**) and 1–3 copies of α-syn from *GAL1p* (**B**); cells were grown in YPD (**A**) and YP-galactose (**B**). Bonferroni post hoc test after a significant two-way ANOVA indicates no significant difference in growth between yeast petites that contain different copies of the α-syn gene. (**C**,**D**) Microscopic images (×400) of petite cells, where expression of 2–3 copies (*MET25p*) or 1–3 copies (*GAL1p*) of α-syn gene was induced, stained with the dye SYTO18. (**E**,**F**) Microscopic images (×400) of petite cells expressing 2–3 copies of α-syn from the *MET25p* (**E**) and 1–3 copies of α-syn from the *GAL1p*, staining with Phloxine B. (**G**,**H**) Western blot analyses of cells expressing 1 to 3-copies of α-syn protein after full induction of the *MET25p* (**G**) or *GAL1p* (**H**). On lanes, 1, 2 (**G**) and 1, 2 and 3 (**H**) were loaded 7.5 µg of total protein obtained after lysis of cells that express 2-copies or 1-copy (lanes 1; **G**,**H**), 3-copies or 2-copies (lanes 2; **G**,**H**) and 3-copies (lane 3; **H**) of α-syn, after the growth of cells under conditions that fully induce the MET25 or GAL1 promoter. The upper panel was probed with an antibody that recognizes human α-syn (Proteintech, #10842-1-AP) and the lower panel with a β-actin antibody (Proteintech, 60008-1-Ig); Densitometric quantification of the α-syn bands in (**G**,**H**) is shown in Supplementary Materials, Parts 3 and 4. Post Hoc Newman-Keuls test, after a significant one-way ANOVA test, indicated a significant difference in cell death, *p* < 0.01, between petites expressing α-syn with different copy numbers.

## Error in Figure 3

In Figure 3A (3C_Met25p), there is a mistake in the white light image. In Figure 3E, there are mistakes in the white and fluorescent light images. In Figure 3F (*1C_GAL1p*), there is a mistake in the white light image.

**Figure 3:**
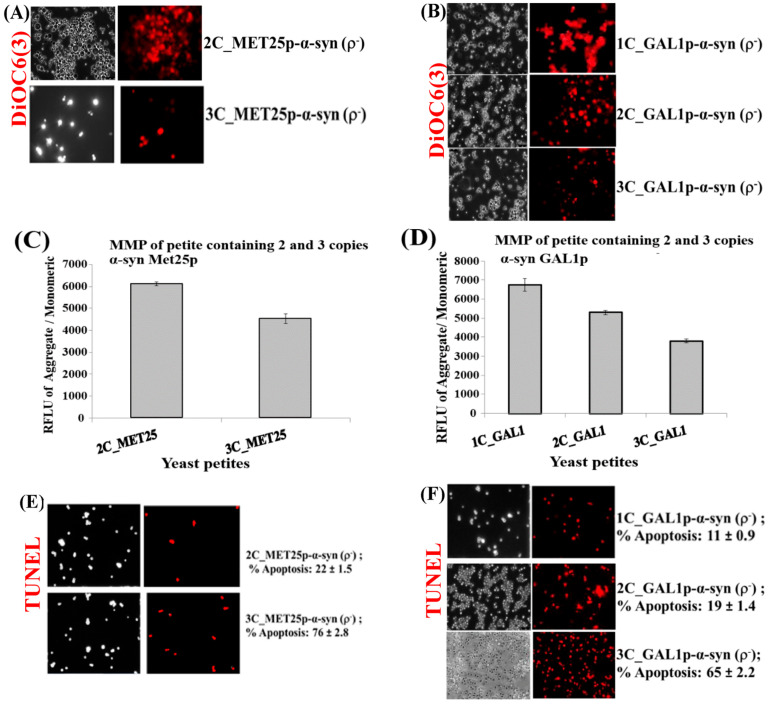
(**A**,**B**) Microscopic images (×400) of staining of 3 copies of α-syn petite transformants with DiOC6(3) dye that detects MMP in live cells. The images are representative images (×400) of cells. (**C**,**D**) Quantification of relative MMP of yeast cells expressing 2 and 3-copies of α-syn from *MET25p* and 1–3-copies of α-syn from *GAL1p*, using the JC-10 dye (*p* < 0.1). (**E**) Microscopic images (×400) of nuclear DNA fragmentation as observed using the TUNEL assay in yeast cells expressing 2 and 3 copies of α-syn from *MET25p*. (**F**) Microscopic images (×400) of nuclear DNA fragmentation as observed using the TUNEL assay in yeast cells expressing 1 copy of α-syn from *GAL1p*. The images are representative images of cells. The data in figures (**C**,**D**) represent mean ± S.D. of three independent experiments (*p* < 0.1; two-tailed *t*-test). The left-hand side pictures in (**A**,**B**,**E**,**F**) show phase-contrast microscopy pictures (×400) of yeast cells. Post Hoc Newman-Keuls test, after a significant one-way ANOVA test, indicated a significant difference *p* < 0.001 between strains expressing 2 and 3-copies of α-syn from *MET25p*, and between 1 and 2-copies and 3-copies of α-syn from *GAL1p*.

## Error in Figure 5

In Figure 5A (*1C_Met25*), there is a mistake in the YPD plate image.

**Figure 5:**
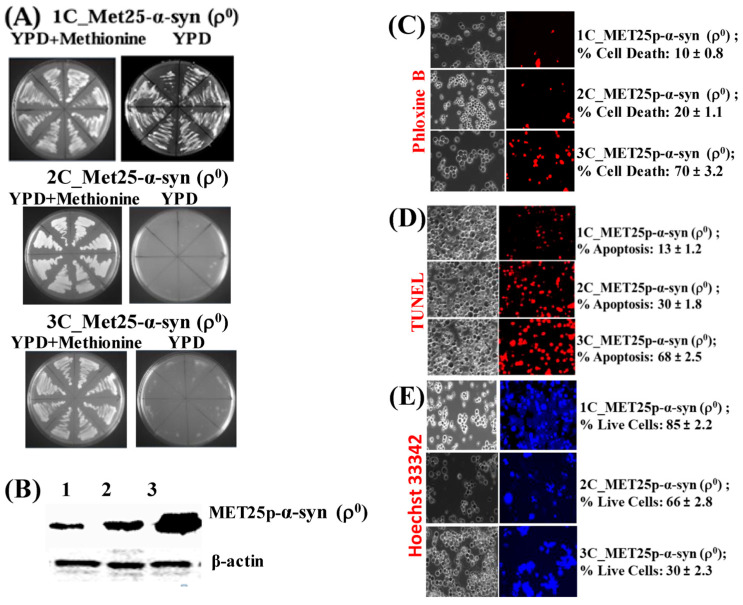
(**A**) Image of growth of Ῥ0 yeast cells harboring 1 copy (1C) of the α-syn gene under *MET25p* control on complete YPD medium agar plates in the presence (YPD + Methionine) or absence of methionine. (**B**) Western blot analyses of ρ^0^ cells expressing 1-copy (lane 1), 2-copies (lane 2), and 3-copies (lane 3) of α-syn protein. On lanes 1, 2, and 3 were loaded 7.5 µg of total protein obtained after lysis of cells. The upper panel was probed with an antibody that recognizes the HA epitope (Proteintech, 51064-2-AP) and the lower panel with a β-actin antibody (Proteintech, 60008-1-Ig); levels of β-actin were used as loading controls, β-actin being a housekeeping gene. (**C**) Microscopic images (×400) of ρ^0^ cells, stained with Phloxine B, after the expression of 1–3 copies of α-syn from the *MET25p*. (**D**) Microscopic images (×400) of nuclear DNA fragmentation, as observed using the TUNEL assay, in ρ^0^ yeast cells expressing 1–3-copies of α-syn from *MET25p*. (**E**) ρ^0^ cells, bearing 1–3 copies of *MET25p*-driven α-syn expression cassettes, were stained with Hoechst 33,342 (a blue dye that labels DNA of live cells). Post Hoc Newman-Keuls test after a significant one-way ANOVA test, indicated a significant difference between petites expressing 1–3 copies of α-syn.

The authors state that the scientific conclusions are unaffected. This correction was approved by the Academic Editor. The original publication has also been updated.
