# Supplementary material for: Apoptosis, Induced by Human α-Synuclein in Yeast, Can Occur Independent of Functional Mitochondria
Source: Cells. 2020 Sep 29;9(10):2203. doi: 10.3390/cells9102203 (PMC7601298; doi:10.3390/cells9102203)
Supplement: Supplementary file 1 [file cells-09-02203-s001.pdf]

cells

MDPI

Apoptosis, induced by human α-synuclein in yeast, can occur independent of functional mitochondria

Damilare D. Akintade [a,b,*] and Bhabatosh Chaudhuri [b]

[a] School of Life Sciences, Medical School, University of Nottingham. NG7 2UH, UK
[b] Leicester School of Pharmacy, De Montfort University, Leicester, LE1 9BH, UK; BChaudhuri@dmu.ac.uk
* Correspondance: Damilare.Akintade@nottingham.ac.uk; Tel.: 07712452922

Contents:

PART 1. Construction of Yeast Strains that Contain 1-copy, 2- and 3-Copies of the Human α-Syn Gene Driven by the *MET25* Promoter (*MET25*p).

PART 2. Construction of Yeast Strains that Contain 1-copy, 2- and 3-Copies of the Human α-Syn Gene Driven by the *GAL1* Promoter (*GAL1*p).

PART 3. Densitometric Quantification of Western Blot Protein Bands after Expression of 2 and 3-Copies of α-Syn Gene from *MET25*p, in ϱ^- Petite Yeast Cells.

PART 4. Densitometric Quantification of Western Blot Protein Bands after Expression of 2 and 3-Copies of α-Syn Gene from *GAL1*p, in ϱ^- Petite Yeast Cells

PART 5. Densitometric Quantification of Western Blot Protein Bands after Expression of 1, 2 and 3-Copies of α-Syn Gene from *MET25*p, in ϱ^0 Petite Yeast Cells.

PART 6. Strain Table

References cited in supporting information.

PART 1: Construction of Yeast Strains that Contain 1-copy, 2- and 3-Copies of the Human α-Syn Gene Driven by the *MET25* Promoter (*MET25*p)

Plasmids were constructed that allow expression of 1-copy, 2- and 3-copies of the α-syn gene in yeast from the *MET25* promoter (*MET25*p). A SpeI-SalI fragment of the coding sequence of the human α-syn gene [NCBI Accession # NM_000345.3] was isolated from a human hippocampal cDNA library (BioCat) by PCR and was cloned in yeast integration vectors downstream of the *MET25*p [*Saccharomyces* Genome Database ID S000004294] and upstream of the CYC1 gene terminator signal [*Saccharomyces* Genome Database ID S000003809]. The *MET25* promoter is repressed in the presence of methionine and induced in its absence. After cloning of the α-syn gene in appropriate yeast integrative vectors, the following plasmids were obtained: YIpHIS3MET25p/alpha-syn (A), YIpURA3MET25p/alpha-syn (B), and YIpTRP1MET25p/alpha-syn (C) (Figure S1). These plasmids contain the α-syn gene sandwiched between the *MET25* promoter and *CYC1* terminator signal and allow integration of 1-copy, 2-copies, and 3-copies of the α-syn gene into chromosomal locations where the auxotrophic markers, *TRP1*, *HIS3*, and *URA3* genes, reside on the yeast genome.

The basic yeast strains used for integration were ϱ^+ W303-1a (*Mata*, *ade2*, *his3*, *leu2*, *trp1*, *ura3*) (ATCC #208352) and its ϱ^0 derivatives generated by treatment with ethidium bromide. Herein, the strains are referred to as BC300 or BC300-ϱ^+ and BC300-ϱ^0. The 1-copy strains contain the *TRP1* integrant. The 2-copy strains contain integrated copies of α-syn at the *TRP1* and *HIS3* loci, whereas the 3-copy strains contain integrated copies of α-syn at the *TRP1*, *HIS3*, and *URA3* loci. To generate negative controls, the strains BC300-ϱ^+ and BC300-ϱ^0 were integrated with empty vectors (i.e., basic integrating vectors which do not contain the α-syn gene) in three successive steps to obtain the 6 strains: (a) BC300::– (TRP1);(b) BC300::– (TRP1),– (HIS3); and (c) BC300::– (TRP1),– (HIS3),– (URA3); (d) BC300-ϱ^0::– (TRP1);(e) BC300-ϱ^0::– (TRP1),– (HIS3); and (f) BC300-ϱ^0::– (TRP1),– (HIS3),– (URA3); they contain one copy, two and three copies of an empty plasmid at the (i) *TRP1*, (ii) *TRP1* and *HIS3*, and (iii) *TRP1, HIS3 and URA3*, chromosomal loci of the strains BC300-ϱ^+ (BC300) and BC300-ϱ^0.

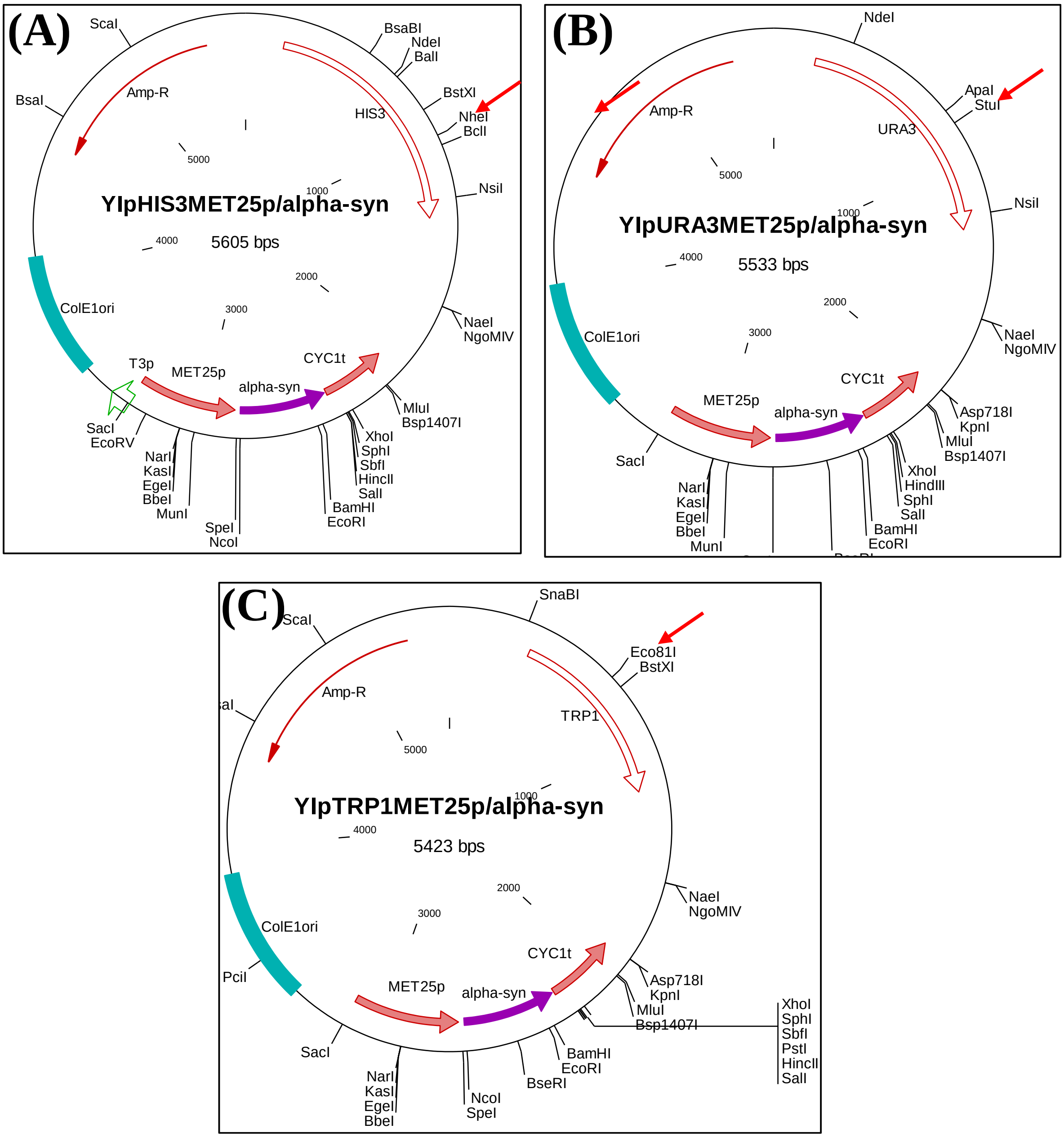

Figure 1. The three integrative plasmids used to introduce human α-syn gene expression cassettes, under the control of the *MET25* promoter, into three different chromosomal locations (i.e., where the *TRP1*, *HIS3*, *URA3* genes lie) of the yeast genome. The arrows show the restriction sites at which the plasmids were linearized for genomic (i.e., chromosomal) integration via homologous recombination [1]. The restriction sites shown occur only once in the plasmid.

<u>PART 2</u>: Construction of Yeast Strains that Contain 1-copy, 2- and 3-Copies of the Human α-Syn Gene Driven by the *GAL1* Promoter (*GAL1*p)

Plasmids were constructed that allow expression of 1-copy, 2- and 3-copies of the α-syn gene in yeast. A BglII-XbaI fragment of the coding sequence of the human α-syn gene [NCBI Accession # NM_000345.3] was isolated from a human hippocampal cDNA library (BioCat) by PCR and was cloned in yeast integration vectors downstream of the GAL1 promoter [*Saccharomyces* Genome Database ID S000000224] and upstream of the SUC2 gene terminator signal [*Saccharomyces* Genome Database ID S000001424]. The GAL1 promoter is repressed in the presence of glucose and induced in the presence of the sugar, galactose After cloning of the α-syn gene in appropriate yeast integrative vectors, the following plasmids were obtained: YIpHIS3GAL1p/alpha-syn (A),

YIpURA3GAL1p/alpha-syn (B), and YIpTRP1GAL1p/alpha-syn (C) (Figure S2). These plasmids contain the α-syn gene sandwiched between the GAL1 promoter and SUC2 terminator signal and allow integration of one copy, two copies and three copies of the α-syn gene into chromosomal locations where the auxotrophic markers, *TRP1*, *HIS3*, and *URA3* genes, reside on the yeast genome.

The basic yeast strain used for integration was BC300. The 1-copy strain contains the *TRP1* integrant. The 2-copy strain contains integrated copies of α-syn at the *TRP1* and *HIS3* loci, whereas the 3-copy strain contains integrated copies of α-syn at the *TRP1*, *HIS3*, and *URA3* loci. To generate negative controls, the strain BC300 was integrated with empty vectors (i.e., basic integrating vectors which do not contain the α-syn gene) in three successive steps to obtain the three strains: (a) BC300::–(TRP1);(b) BC300::– (TRP1),– (URA3); and (c) BC300::– (TRP1),– (HIS3),– (URA3); they contain one copy, two and three copies of an empty plasmid at the (i) *TRP1*, (ii) *TRP1* and *HIS3*, and (iii) *TRP1, HIS3 and URA3*, chromosomal loci.

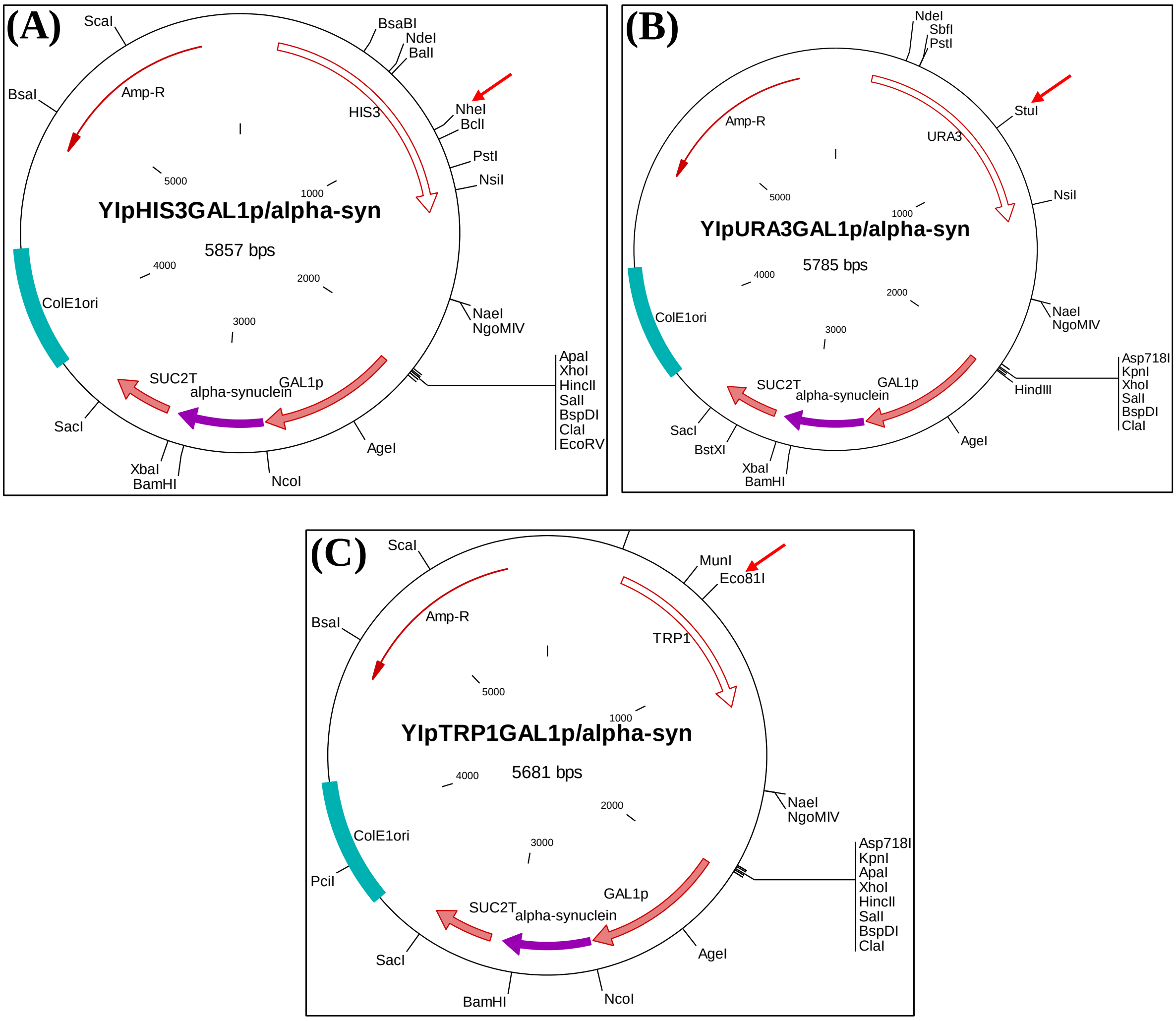

Figure 2. The three integrative plasmids used to introduce human α-syn gene expression cassettes, under the control of the *GAL1* promoter, into three different chromosomal locations (i.e., where the TRP1, HIS3, URA3 genes lie) of the yeast genome. The arrows show the restriction sites at which the plasmids were linearized for genomic (i.e., chromosomal) integration via homologous recombination [1]. The restriction sites shown occur only once in the plasmid.

<u>PART 3</u>: Densitometric Quantification of Western Blot Protein Bands after Expression of 2- and 3-Copies of α-Syn Gene from *MET25*p, in ϱ^- Petite Yeast Cells

This densitometric quantification represents the Western blot depicted in Figure 2G of the manuscript. It clearly shows that the 3-copy strain expresses higher levels of α-syn than the 2-copy strain.

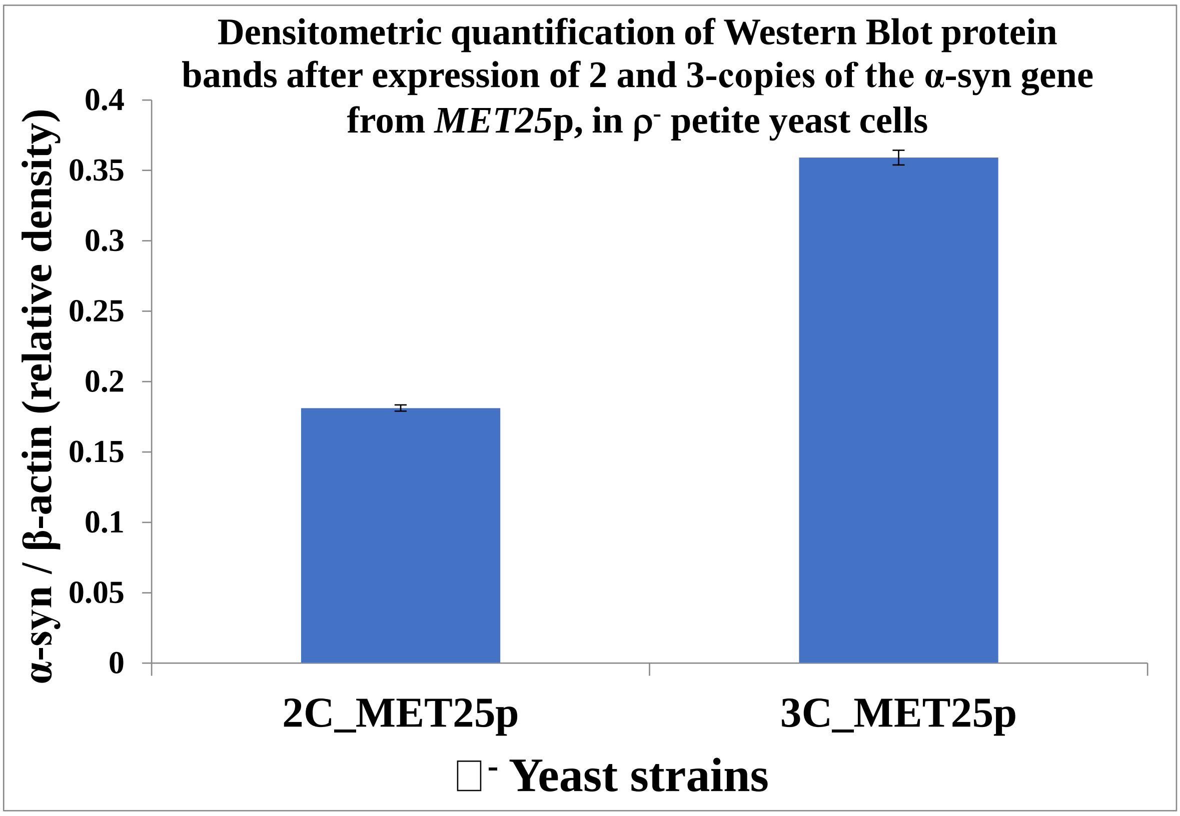

Figure 3. Densitometric quantification of Western Blot bands (as in Figure 2G) that show expression of α-syn protein from cells that express of 2, 3 copies of the α-syn gene from the *MET25* promoter in ϱ^- petite yeast cells. The values for β-actin (not shown) were roughly the same. The values represent the average of three independent experiments ($p<0.05$).

<u>PART 4</u>: Densitometric Quantification of Western Blot Protein Bands after Expression of 2 and 3-Copies of α-Syn Gene from *GAL1*p, in ϱ^- Petite Yeast Cells

This densitometric quantification represents the Western blot depicted in Figure 2H of the manuscript. It shows that there is a gradual increase of α-syn protein levels expressed in cells harbouring 1 to 3-copies of the α-syn gene.

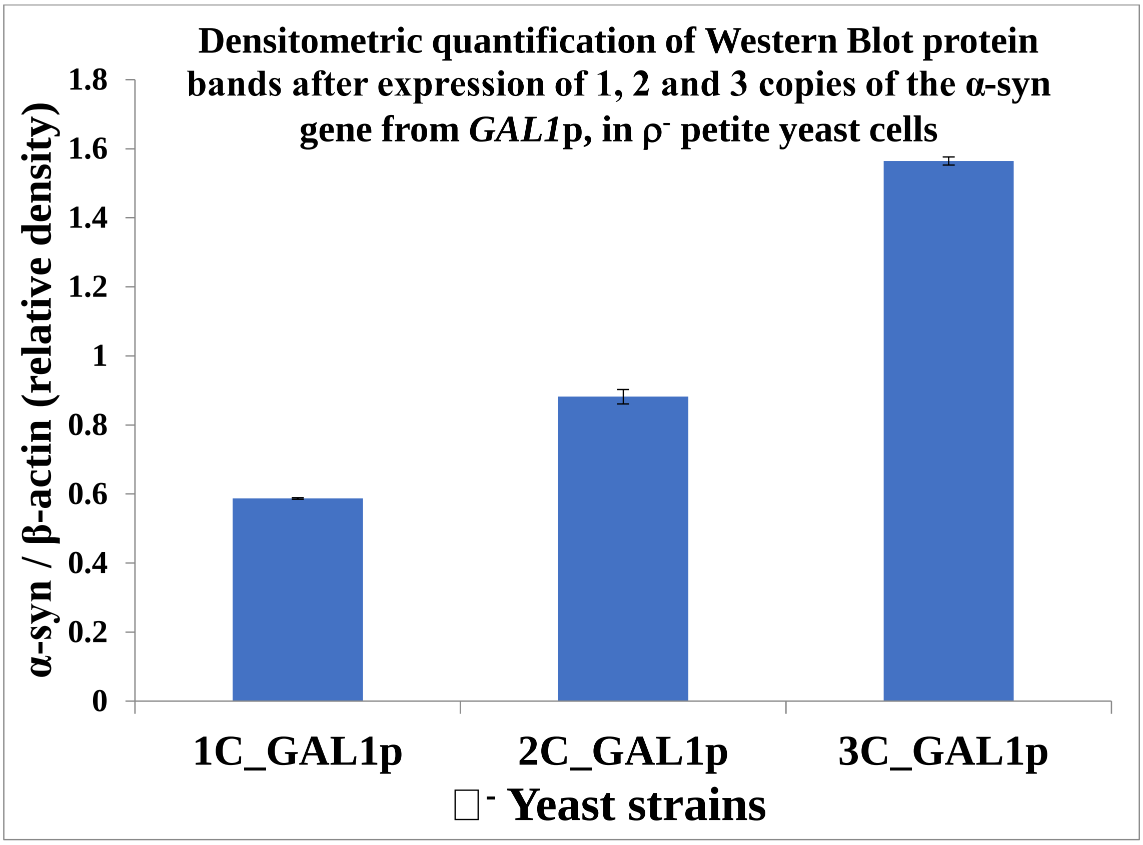

Figure 4. Densitometric quantification of Western Blot bands (as in Figure 2H) that show expression of α-syn protein from cells that express of 1, 2, 3 copies of the α-syn gene from the *GAL1* promoter in ϱ- petite yeast cells. The values for β-actin (not shown) were roughly the same. The values represent the average of three independent experiments ($p<0.05$).

PART 5: Densitometric Quantification of Western Blot Protein Bands after Expression of 1, 2 and 3-Copies of α-Syn Gene from *MET25*p, in ϱ⁰ Petite Yeast Cells

This densitometric quantification represents the Western blot depicted in Figure 5B of the manuscript. It again shows that the 3-copy strain expresses nearly twice as much α-syn as the 2-copy strain.

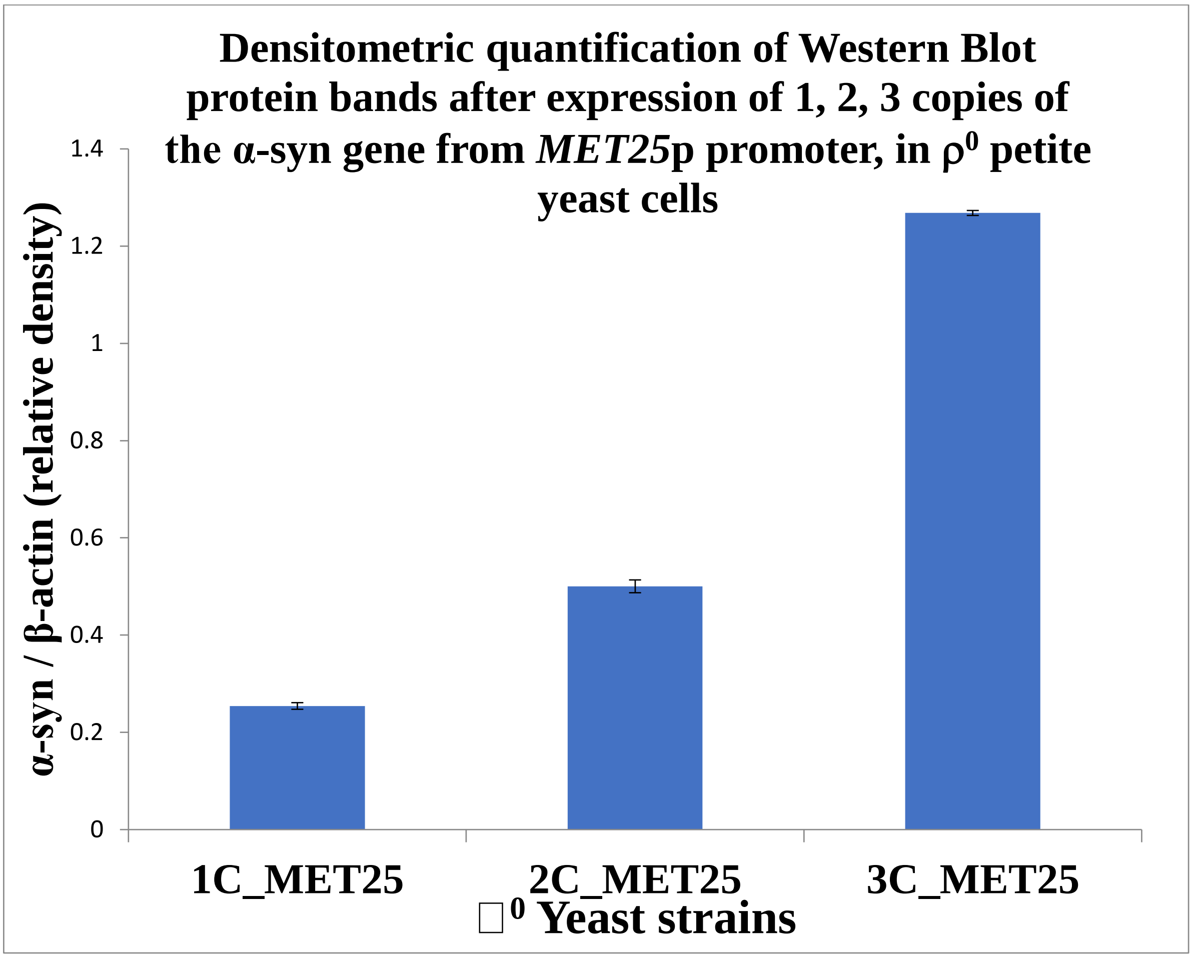

Figure 5. Densitometric quantification of Western Blot bands (as in Figure 5B) that show expression of α-syn protein from cells that express of 1, 2, 3 copies of the α-syn gene from the *MET25* promoter in ϱ^0 petite yeast cells. The values for β-actin (not shown) were roughly the same. The values represent the average of three independent experiments ($p<0.05$).

<u>PART 6:</u> Strain table

No	Strains (With α-Syn Copy No)	Genotypes (Loci Where α-Syn Was Integrated)
1	Rho+ BC300 1C (GAL1p-α-Syn)	(TRP1)
2	Rho+ BC300 2C (GAL1p-α-Syn)	(TRP1),– (HIS3)
3	Rho+ BC300 3C (GAL1p-α-Syn)	(TRP1),– (HIS3),– (URA3)
4	Rho- BC300 1C (GAL1p-α-Syn)	(TRP1)
5	Rho- BC300 2C (GAL1p-α-Syn)	(TRP1),– (HIS3)
6	Rho- BC300 3C (GAL1p-α-Syn)	(TRP1),– (HIS3),– (URA3)
7	Rho0 BC300 1C (GAL1p-α-Syn)	(TRP1)
8	Rho0 BC300 2C (GAL1p-α-Syn)	(TRP1),– (HIS3)
9	Rho0 BC300 3C (GAL1p-α-Syn)	(TRP1),– (HIS3),– (URA3)
10	Rho+ BC300 1C (Met25p-α-Syn)	(TRP1)
11	Rho+ BC300 2C (Met25p-α-Syn)	(TRP1),– (HIS3)
12	Rho+ BC300 3C (Met25p-α-Syn)	(TRP1),– (HIS3),– (URA3)
13	Rho- BC300 2C (Met25p-α-Syn)	(TRP1),– (HIS3)
14	Rho- BC300 3C (Met25p-α-Syn)	(TRP1),– (HIS3),– (URA3)
15	Rho0 BC300 1C (Met25p-α-Syn)	(TRP1)
16	Rho0 BC300 2C (Met25p-α-Syn)	(TRP1),– (HIS3)
17	Rho0 BC300 3C (Met25p-α-Syn)	(TRP1),– (HIS3),– (URA3)

Reference

1. Joska, T.M.; Mashruwala, A.; Boyd, J.M.; Belden, W. J. A universal cloning method based on yeast homologous recombination that is simple, efficient, and versatile. *J. of microb. Met*. **2014**, *100*, 46–51.

© 2020 by the authors. Licensee MDPI, Basel, Switzerland. This article is an open access article distributed under the terms and conditions of the Creative Commons Attribution (CC BY) license (http://creativecommons.org/licenses/by/4.0/).
